# Duplex kidney complicated with preoperative inferior nephroblastoma rupture in children: a case report and literature review

**DOI:** 10.1186/s12887-021-02919-2

**Published:** 2021-10-08

**Authors:** Yongxiang Zhao, Haiyan Cheng, Hongcheng Song, Ruimin Zhang, Xiangming Wu, Haowei Li, Jun Wang, Huanmin Wang, Chunmei Jia

**Affiliations:** 1 The Fourth Hospital of Baotou, Baotou, China; 2 Beijing Children’s Hospital, No, 6 Nanlishi Road, Xicheng District, Beijing, 100000 China

**Keywords:** Children, Nephroblastoma, Duplex kidney, Rupture

## Abstract

**Background:**

We admitted a child with a duplex kidney combined with preoperative rupture of nephroblastoma and used this case to discuss the clinical features and treatment of this disease.

**Case presentation:**

We retrospectively analyzed the clinical data of a 5-year-old girl with preoperative duplex kidney rupture combined with inferior nephroblastoma who was admitted to the Fourth Hospital of Baotou. In addition, we reviewed the relevant literature. The patient’s details were as follows: weight, 17 kg; height, 108 cm; and body surface area, 0.7 m^2^. Abdominal ultrasound for abdominal pain revealed the presence of a left-sided renal mass; enhanced abdominal computed tomography further confirmed it to be a left-sided duplex kidney measuring approximately 6 × 5 × 5 cm, with a rupture originating from the lower kidney. The PubMed database was searched from 2010 to 2020 for the terms “Wilms’ tumor” and “Duplex” and “Wilms’ tumor” and “Rupture.” The treatment plan was preoperative chemotherapy (vincristine/dactinomycin, VA regimen) + left kidney tumor radical surgery + postoperative chemotherapy (actinomycin-D/VCR/doxorubicin, AVD regimen). Postoperative pathology revealed an International Society of Pediatric Oncology intermediate-risk stage-3 nephroblastoma (mixed type) in the left kidney. Literature review was performed with 71 cases meeting the set criteria with an aim to analyze and summarize the clinical characteristics and treatment of patients with ruptured nephroblastoma and duplex kidney combined with nephroblastoma.

**Conclusions:**

To our knowledge, no previous studies have reported preoperative duplex kidney combined with nephroblastoma rupture. In patients with this condition, preoperative chemotherapy is recommended when the vital signs are stable and tumor resection can be performed after the tumor has shrunk to prevent secondary spread. If the patient’s vital signs are unstable, emergency exploratory surgery is needed. If the nephroblastoma rupture is old and limited, surgery can be performed when the tumor size is small.

## Background

Nephroblastoma (Wilms’ tumor [WT]) is the most common pathological type of renal tumors in children, accounting for approximately 85% of renal tumors in children [1]. Rupture can be caused by large tumors or trauma, with an incidence of approximately 2–5%. Although duplex kidney deformity is also a common kidney malformation [2], duplex kidney with nephroblastoma has rarely been reported. Here we report the diagnosis and management of a child admitted to our hospital owing to the preoperative rupture of a left duplex kidney combined with inferior nephroblastoma.

## Case presentation

**Fig. 1:**
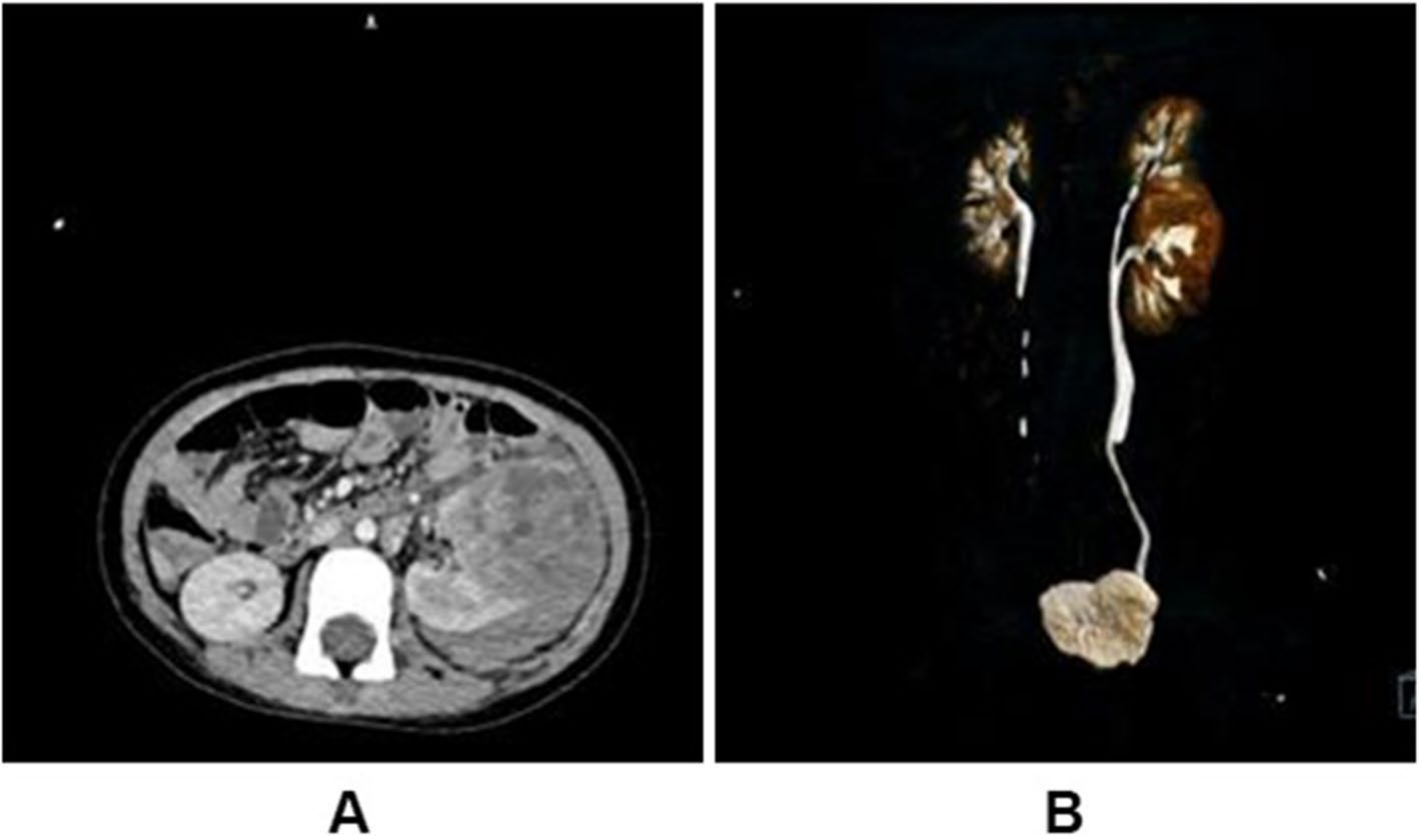
**A** Before chemotherapy, the normal morphology of the patient’s left kidney was absent on cross-sectional computed tomography (CT) scan, with an irregular soft-tissue mass, a slightly higher-density shadow of approximately 4.5 × 4.7 × 5.6 cm in size, double renal pelvis of the left kidney, and confluence of the two ureters at the entrance of the pelvis. **B** Before the patient’s chemotherapy, the normal morphology of the left kidney disappeared on the coronal CT urography scan. The visible size is of approximately 4.5 × 4.7 × 5.6 cm; irregular soft group weave a block shadow and a slightly higher-density shadow; the left kidney and renal pelvis; the two ureters meet at the entrance of the pelvic cavity

**Fig. 2:**
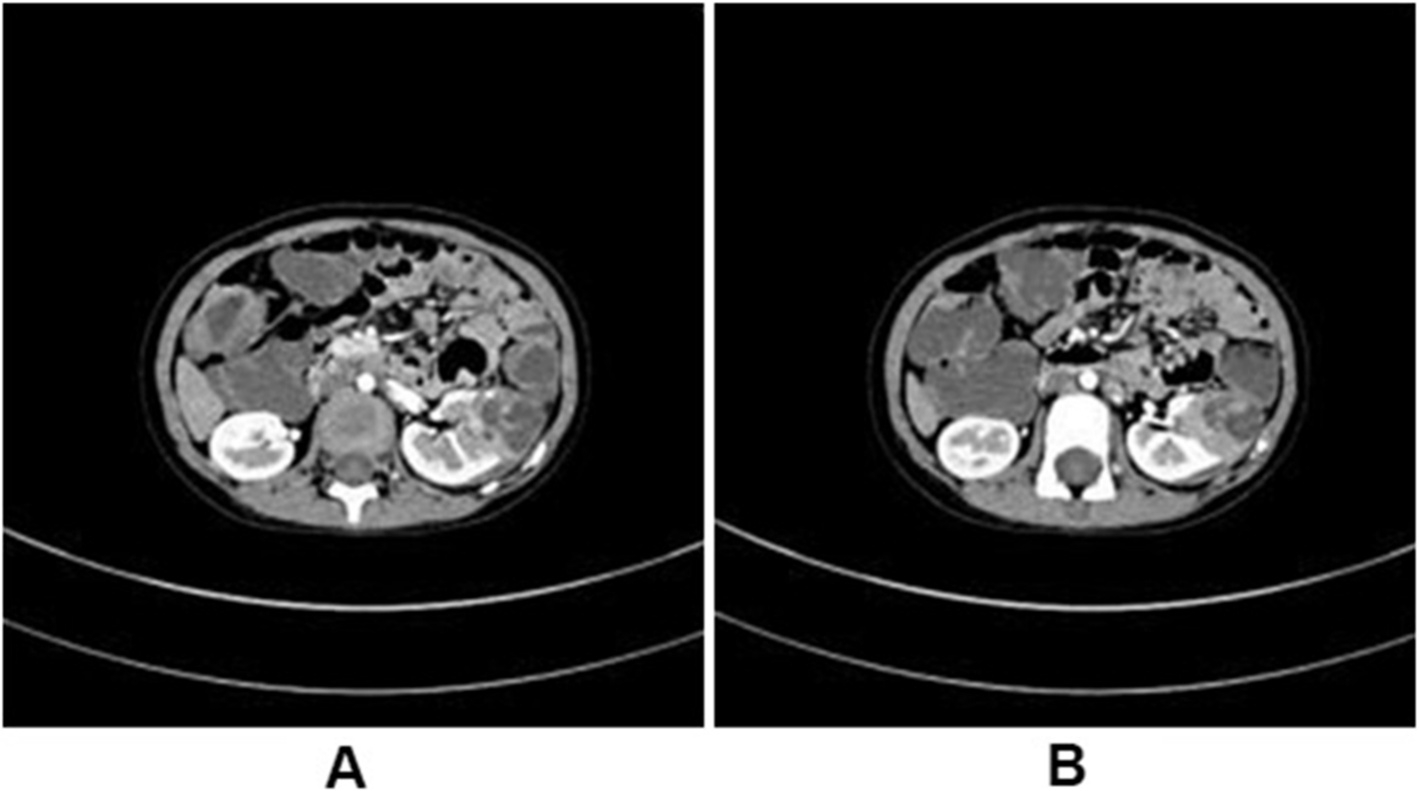
Different sections of the computed tomography scan after chemotherapy revealed an approximately 3.1 × 3.2 × 4.2 cm irregular mass in the left renal parenchyma, with the shadows of irregular soft-tissue masses

The patient’s details were as follows: sex, female; age, 5 years and 1 month; weight, 17 kg; height, 108 cm; and body surface area, 0.7 m^2^. Abdominal ultrasound for abdominal pain revealed a tumor in the left kidney. The tumor was approximately 6 × 5 × 5 cm and primarily solid in texture; the patient had no history of trauma. Further abdominal enhanced computed tomography (CT) revealed (Fig. 1) that the left kidney was a duplex kidney; the tumor originated from the lower kidney and sized approximately 4.5 × 4.7 × 5.6 cm, with slight enhancement; and the CT value was approximately 32–41 Hu. Multiple effusions were observed around the kidney and pelvis. Given that the renal tegument was locally incomplete, tumor rupture was considered. No metastatic lesions were found on head or chest CT, and the renal function was normal. The hemoglobin level was 104 g/L, and the patient’s vital signs were stable. Owing to the tumor rupture, it was treated with vincristine (VCR) plus dactinomycin (VA regimen) for 4 weeks before surgery. The tumor shrunk; an enhanced CT scan revealed (Fig. 2) that the tumor in the left renal parenchyma was smaller than before—approximately 3.1 × 3.2 × 4.2 cm.

**Fig. 3:**
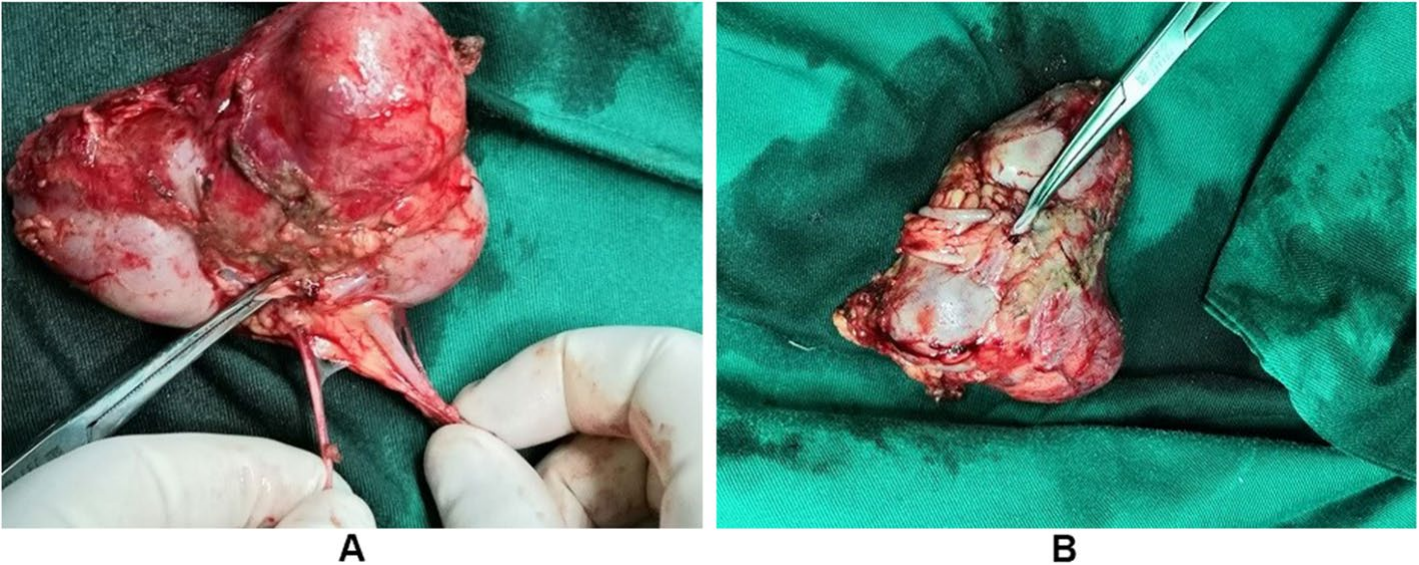
**A** Surgical specimens after the radical resection of the renal tumor; the duplex kidney and duplex ureter can be observed. The tumor size is 3.1 × 3.2 × 4.2 cm. **B** Surgical specimens after radical nephrectomy; repeated kidney malformations and nephroblastoma can be noted. The tumor size is 3.1 × 3.2 × 4.2 cm

**Fig. 4:**
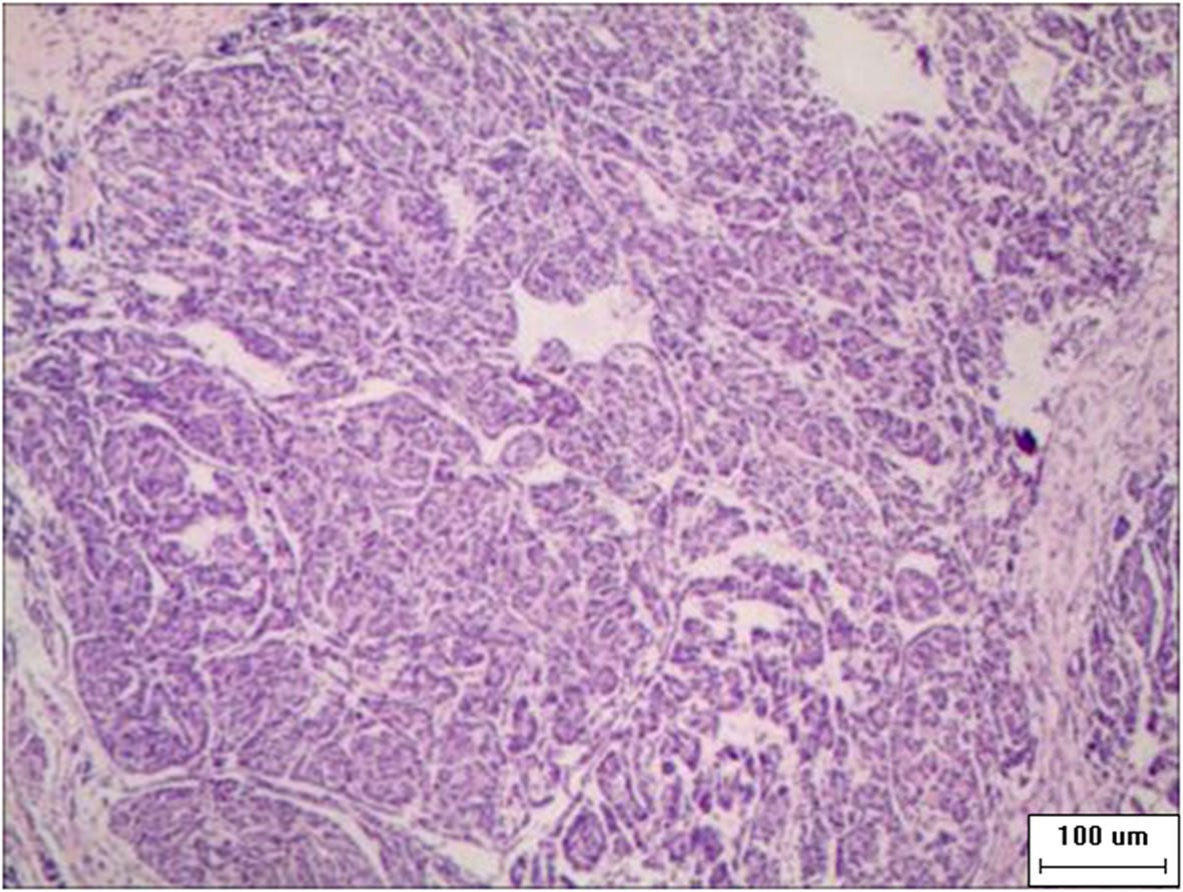
Postoperative pathological image (Zeiss Axio Scope A1,Mingmei Digital Imaging System MD50-B, HE staining, 10 × 10): left nephroblastoma (mixed type). Postoperative pathology: left nephroblastoma (hybrid)

After preoperative preparation, radical resection of the left renal tumor was performed under general anesthesia. During the surgery, the left kidney was confirmed to be a duplex kidney and the tumor originated from the middle pole of the lower kidney, sized approximately 3 × 3 × 4 cm, with irregular morphology. An old rupture could be observed at the dorsal side, adhered to the surrounding tissues and the psoas major muscle. The tumor was dissociated from the renal pedicle from the lateral, lower, and medial sides by blunt combined with sharp dissociation. The renal pedicle was ligated, and the tumor was completely resected (Fig. 3). Eight pieces of renal hilum and para-aortic lymph nodes were taken for pathological examination. Postoperative pathological examination (Beijing Children’s Hospital) revealed the presence of a left mixed-type nephroblastoma (Fig. 4); 20% tumor bleeding and necrosis; invasion of tumor tissues to the renal capsule, (renal sinus) adipose tissues, renal pelvis, and ureter; and absence of tumor infiltration to the renal hilum and retrocapsular lymph nodes (in turn, 0/8, 0/2). The immunohistochemical results showed WT1 (focal+), cytokeratin CK (AE1/AE3) (+), Ki-67 (20%), CD10 (scattered+), CD56 (+), synaptophysin (+), myogenin (−), and desmin (−); The International Society of Pediatric Oncology (SIOP) staging was intermediate-risk stage 3. Postoperative SIOP chemotherapy (actinomycin-D/VCR/doxorubicin [DOX]) was performed for 30 weeks. The patient was followed up for 11 months, and no tumor recurrence or metastasis was noted.

### Literature

The PubMed database was searched from 2010 to 2020 for the terms “Wilms’ tumor” and “Duplex” and “Wilms’ tumor” and “Rupture.” Two cases of WT combined with duplex kidney have been reported in the literature in the last decade [3, 4], which combined with our case makes a total of three cases reporting this condition. A total of 69 cases of WT combined with rupture have been reported in the literature [5, 6]; thus, we analyzed a total of 70 cases (69 plus our case), which are presented below.

### Nephroblastoma combined with duplex kidney

General data of the three patients (including our patient) with WT combined with duplex kidney are shown in Table 1. The patients were aged 10, 4, and 5 years. These children presented with abdominal pain in two cases and intermittent fever in one case. Preoperatively, all were diagnosed with duplex kidney combined with inferior renal tumor on abdominal enhancement CT. One patient was treated with preoperative chemotherapy + surgery + postoperative chemotherapy, and the other two were treated with surgery + postoperative chemotherapy. The postoperative pathological examination revealed a stage-1/2/3 good-prognosis-type nephroblastoma in each patient.

**Table 1:** General profiles of WT patients with duplex kidney reported in the literature in the past ten years

NO.	Author	Year	Patient age (years)	Number of patients	sex	Tumor size (cm)	Location	Treatment options	Duration of chemotherapy	Chemotherapy drugs	Staging
1	Ibrahim Karnak et al. [4]	2017	10	1	F	–	Right side	Radical nephrectomy + post-operative chemotherapy	6 Months	–	Phase II
2	Abdol-Mohammad Kajbafzadeh et al. [3]	2013	4	1	M	8 × 7 × 3.5	Left side	Radical nephrectomy + post-operative chemotherapy	9 Months	ACTD+VCR	Good histology confined to the renal envelope
3	The present example	2020	5	1	F	3.1 × 3.2 × 4.2	Left side	Preoperative chemotherapy + radical nephrectomy + postoperative chemotherapy	30 weeks	SIOP (AVD) chemotherapy regimen (ACD/VCR/DOX)	SIOP stage III intermediate risk

Combined rupture of nephroblastoma

General data of the 70 patients (including our patient) with combined WT rupture are shown in Table 2. Patient details were as follows: age at diagnosis, 2 days–12 years; mean age at diagnosis, 45 months; condition combined with inferior vena cava aneurysm embolism, 4; and treatment, radical nephrectomy + postoperative radiotherapy: 13 and preoperative chemotherapy + radical nephrectomy + postoperative radiotherapy: 57. A total of 13 (13/70, 18.6%) patients had recurrence and metastasis, and 9 (9/70, 12.9%) died within 0.2–16.3 years of follow-up.

**Table 2:** Ruptured nephroblastoma

NO.	Author	Year	Patient age (mean)	Number of patients	Sex (M:F)	Tumor size	Location (L:R)	Treatment options	Clinical manifestations and complications	Type of pathology
1	Marie-Amelyne Le Rouzic et al. [7]	2019	4 years	28	12:16	≤400 ml: 7 ≥400 ml: 20	12:16	Preoperative chemotherapy + surgery + postoperative chemotherapy	Recurrence: 3 Death: 2	Epithelial type: 1 Mixed type: 9 Regressive type: 12 Blastemal type: 5 Diffuse anaplastic type:1
2	Zhang Y et al. [8]	2020	42.5 ± 18.7 Months	41	24: 17	Average diameter:12 cm Volume:533.6 cm^3^	20:21	Immediate group (immediate surgery without preoperative chemotherapy): 13 Delayed group (delayed surgery after preoperative chemotherapy): 28	Immediate group: Partial rupture: 12 Continuous bleeding: 1 Recurrence and metastasis: 2 Delayed group: Inferior vena cava tumor embolism: 4 Recurrence and metastasis: 8 Death: 7	Immediate group: Blastemal type: 5 Mixed type: 8 Delayed group: Blastemal type: 5 Mixed type: 10 Epithelial type: 1 Stromal type: 2 Regressive type: 3 completely necrotic: 2 fetal rhabdomyoma nephroblastoma: 1 non-typable: 4
3	The present example	2020	5 years and 1 month	1	0:1	3.1 × 3.2 × 4.2 cm	1:0	chemotherapy + surgery + postoperative chemotherapy	–	Mixed type: 1

## Discussion and conclusions

Nephroblastoma (WT), which is relatively common in children, accounts for 6–7% of malignant tumors in children aged < 15 years; of this, approximately 75% occur in children aged < 5 years [1]. Renal duplex malformation is a common type of kidney malformation in children, which can be of two types based on the condition of the ureter: complete and Y-type duplexes. The occurrence of nephroblastoma might be associated with persistent metanephric blastema; however, the exact etiology is unknown and is considered to be associated with heredity and gene mutation. Known gene mutations include *WT1*, *WTX*, *p53*, and *1P16Q*. Horseshoe kidney has been reported to be a complication of nephroblastoma [9]; however, to our knowledge, no previous studies have reported Duplex kidney complicated with nephroblastoma rupture. The literature presents only two cases of duplex kidney complicated with nephroblastoma till date [3, 4].

Preoperative nephroblastoma rupture is relatively rare. Among the 606 cases of nephroblastoma reported by Lucian L [10], 19 (3%) tumors were found to have ruptured preoperatively. When the tumor ruptures with a small fissure and bleeding, children may present with abdominal pain accompanied by nausea, vomiting, appetite loss, and other symptoms, most of which can resolve without any treatment. However, when the tumor ruptures with a large fissure and bleeding, children may present with sudden and severe abdominal pain accompanied by vomiting, obvious tenderness of the whole abdomen, muscle tension, and even shock or other clinical manifestations. To summarize, in the 70 patients with preoperative WT rupture, abdominal pain was the predominant clinical manifestation in 44 (44/70, 62.8%) patients, followed by decreased hemoglobin in 21 (21/70, 30%); only 11 (11/70, 15.7%) patients had a clear history of trauma. The diagnosis of WT rupture on abdominal CT has high specificity but relatively low sensitivity [11]; 15 (15/70, 21.4%) patients did not show clear imaging changes. Therefore, combining clinical symptoms with imaging is necessary for a comprehensive diagnosis. Our patient had no history of trauma, and abdominal enhanced CT revealed multiple perirenal and pelvic effusions, indicating tumor rupture. Only the preoperative hemoglobin levels were slightly lower than the normal, and other vital signs were stable. During the surgery, the rupture of the tumor was confirmed.

After detecting the preoperative rupture of the nephroblastoma, we were unsure regarding whether to operate before or after chemotherapy. A review of the literature revealed that acute tumor rupture and massive bleeding may result in hemodynamic changes, which may lead to an acute hemorrhagic shock and is life-threatening. Emergency surgery is needed for those who receive ineffective conservative treatment. During the surgery, the bleeding should be stopped completely and the tumor should be removed maximally [12]. If the nephroblastoma is ruptured but the vital signs are stable, preoperative chemotherapy can be performed and the surgery can be performed when the tumor has shrunk and the rupture is limited to reduce the possibility of secondary contamination of the abdominal cavity during the surgery [13]. Of the 70 patients with combined rupture of WT, 13 had old and limited ruptures and a small tumor; they were treated with radical nephrectomy and postoperative chemotherapy. A total of 57 patients were treated with preoperative chemotherapy + radical nephrectomy, and all were treated with conventional radiotherapy after surgery. Only two cases of recurrence and metastasis occurred among the patients who underwent only radical nephrectomy, whereas 11 cases of recurrence and metastasis and 9 deaths occurred in those who received preoperative chemotherapy. The different inclusion criteria and the facts that the tumors in the patients who underwent only radical nephrectomy were relatively small, the rupture was more limited, and the tumors could be completely resected might explain the difference. Before surgery, our patient presented with only abdominal pain. Her vital signs were stable, and she had no abdominal muscle tension or other acute abdominal symptoms; thus, preoperative VA-regimen chemotherapy was administered for 4 weeks and the tumor size reduced significantly. During the surgery, we confirmed that the old rupture at the dorsal side of the tumor had adhered to the surrounding tissues but the collapse was limited and the tumor was removed completely during the operation.

Owing to the low number of related reports, whether partial nephrectomy is feasible in cases of duplex kidney complicated with nephroblastoma needs to be explored further. Cost [14] believed that nephron sparing surgery (NSS) may be feasible for specific patients with unilateral WT. NSS has been reported to be feasible if the tumor is located at one pole of the kidney, it is small (generally < 4 cm) with duplex kidney, and the residual kidney size is > 50% [7]. However, we did not perform NSS in our patient because the tumor was ruptured preoperatively. For duplex kidney complicated with inferior nephroblastoma and preoperative rupture, care should be taken to avoid further spread of the rupture during surgery. The rupture would be limited after preoperative chemotherapy; however, at the same time, the ruptured tumor might adhere to surrounding tissues or organs; therefore, it should be removed carefully, leaving no residual tumor tissue and causing no damage to the surrounding organs. When dealing with the renal pedicle, attention should be paid to the vascular supply of the upper and lower parts of the duplex kidney. If excessive blood vessels are noted, the renal pedicle can be ligated several times to prevent the ligation line from falling off or being loose.

Preoperative rupture of nephroblastoma is a factor that influences disease prognosis [8]. The recurrent metastasis rate was high at 18.6% in the 70 patients with combined WT rupture. Our patient was postoperatively treated with VA regimen and DOX and was closely monitored and followed up. Thus far, she has been followed up for 11 months and no tumor recurrence or metastasis has been noted.

In conclusion, to our knowledge, the preoperative rupture of duplex kidney combined with nephroblastoma has not been reported yet. We believe that preoperative chemotherapy is the first treatment option to reduce the tumor size and define the extent of rupture and that an immediate surgery is also an option for patients with hemorrhagic shock or relatively small tumor size with limited rupture. Owing to the small number of clinical cases reported in the literature, further studies are warranted to determine whether the kidney can be preserved for NSS in duplex kidney combined with inferior nephroblastoma. Preoperative chemotherapy is feasible when the nephroblastoma ruptures preoperatively and the vital signs are stable, and tumor resection can be performed after limited rupture to prevent secondary spread of the tumor. Preoperative tumor rupture affects the disease prognosis; thus, triple chemotherapy and radiotherapy should be initiated after surgery if necessary.

## Data Availability

The datasets used and/or analyzed during the current study are available from the corresponding author on reasonable request.

## Abbreviations

CT: Computed tomography
WT: Wilms’ tumor
NSS: Nephron sparing surgery
TN: Total nephrectomy
